# Physique and Performance of Young Wheelchair Basketball Players in Relation with Classification

**DOI:** 10.1371/journal.pone.0143621

**Published:** 2015-11-25

**Authors:** Valentina Cavedon, Carlo Zancanaro, Chiara Milanese

**Affiliations:** Department of Neurologicaland Movement Sciences, University of Verona, Verona, Italy; Research Center for Sports Sciences, Health and Human Development (CIDESD), University of Trás-os-Montes e Alto Douro, Vila Real, Portugal, PORTUGAL

## Abstract

The relationships among physical characteristics, performance, and functional ability classification of younger wheelchair basketball players have been barely investigated to date. The purpose of this work was to assess anthropometry, body composition, and performance in sport-specific field tests in a national sample of Italian younger wheelchair basketball players as well as to evaluate the association of these variables with the players’ functional ability classification and game-related statistics. Several anthropometric measurements were obtained for 52 out of 91 eligible players nationwide. Performance was assessed in seven sport-specific field tests (5m sprint, 20m sprint with ball, suicide, maximal pass, pass for accuracy, spot shot and lay-ups) and game-related statistics (free-throw points scored per match, two- and three-point field-goals scored per match, and their sum). Association between variables, and predictivity was assessed by correlation and regression analysis, respectively. Players were grouped into four Classes of increasing functional ability (A-D). One-way ANOVA with Bonferroni’s correction for multiple comparisons was used to assess differences between Classes. Sitting height and functional ability Class especially correlated with performance outcomes, but wheelchair basketball experience and skinfolds did not. Game-related statistics and sport-specific field-test scores all showed significant correlation with each other. Upper arm circumference and/or maximal pass and lay-ups test scores were able to explain 42 to 59% of variance in game-related statistics (P<0.001). A clear difference in performance was only found for functional ability Class A and D. Conclusion: In younger wheelchair basketball players, sitting height positively contributes to performance. The maximal pass and lay-ups test should be carefully considered in younger wheelchair basketball training plans. Functional ability Class reflects to a limited extent the actual differences in performance.

## Introduction

Wheelchair basketball (WB) represents one of the most popular sports for the disabled. Worldwide, it is played at a competitive level in nearly hundred countries and it has grown to about 30,000 participants. In the last several years there has been an increased understanding by spectators, an increase in the number of organisations, a greater understanding of the physical training and coaching, and the creation of a well-developed classification system of the athletes [1,2,3]. Wheelchair basketball is played under the jurisdiction of the International Wheelchair Basketball Federation (IWBF; www.iwbf.org) that has the mandate of establishing the Official Wheelchair Basketball Rules, the Official Wheelchair Basketball Player Classification Handbook, the specifications for equipment and facilities as well as the Internal Regulations that govern the conduct of the Federation. The IWBF rules the world championships for senior men and senior women (i.e., the Gold Cup), as well as for junior men (i.e., under-23) and junior women (i.e., under-25). The IWBF states that WB is designed for athletes with a permanent physical impairment resulting in lower limb physical limitation (e.g., spinal cord injury, amputations, joint and musculoskeletal conditions) that prevent running, jumping and pivoting at speed and with the control, safety, stability and endurance of an able-bodied player. Players who meet the above mentioned eligibility criteria are assigned functional points from 1.0 point (minimal functional potential) through to 4.5 points (maximal functional potential), on an ordinal scale. Wheelchair basketball retains most major rules and scoring of the sport of running basketball (for example, a 10-foot basketball hoop and standard basketball court), but introduces some adaptations in consideration of the presence of subjects with different impairments (by having a classification system of players and a rule of team balance) and the use of the wheelchair in the game (e.g. ‘travelling’ in wheelchair basketball occurs when the athlete touches his/her wheels more than twice after receiving or dribbling the ball).

Wheelchair basketball is an intermittent activity demanding simultaneously several skills, for wheelchair manoeuvring (i.e., propulsion, starting and stopping and changing direction of the wheelchair) and ball handling (i.e., shooting, passing, dribbling or rebounding) [4]. Abundant information is available on WB performance of adult male and female players focusing on the classification system, field tests, performance analysis as well as physiological, biomechanical, technical and tactical aspects [2,3,5–13]. However, the physical and performance characteristics of younger WB players have not been given attention. In fact, a literature research performed in December 2013 using the electronic database PubMed (keywords: wheelchair; basketball) yielded 111 papers, none of which specifically dealt with the physical and performance characteristics of younger WB players. On the other hand, WB represents one of the fastest growing sports for young male and female with physical impairments, and it is played by young players in several countries around the word. For example, in the last edition of the IWBF World Wheelchair Basketball Championships for men (under-23; Turkey 2013) and women (under-25; Canada 2011), twelve and eight national teams were present, respectively. Accordingly, research is needed to characterize younger WB players. In order to increase youth WB participation and to create competitive opportunities for young and aspiring athletes, a number of countries members of the IWBF (e.g. USA, Great Britain, Italy, Canada and Australia) have, further to the men’s under-23 and women’s under-25 national teams, national championships for younger players. The technical and medical regulations governing such championships are often set out by the national WB federations under the surveillance of the IWBF and may vary across countries.

For example, in Italy, younger WB players (≤22 y) are admitted to compete in the Italian Young Wheelchair Basketball Championship. This championship is managed by the Federazione Italiana Pallacanestro in Carrozzina (Italian Wheelchair Basketball Federation), FIPIC [14]. The technical and medical regulations governing the championship are set out by the FIPIC under the surveillance of the Italian Paralympic Committee, the International Paralympic Committee, and the IWBF. In the Italian Young Wheelchair Basketball Championship the height of the basket is lower (2.6m) with respect to the Italian premier league (A1), A2, and B league and the ball size is reduced (weight: 400–500 g, circumference: 68–73 cm). Athletes are classified according to the IWBF Official Player Classification Manual (i.e., players are assigned 1.0–4.5 functional points according to functional potential). However, points are added or subtracted to each athlete on the basis of sex and play experience: 2 points are subtracted from female players; players of either sex who have been playing WB for less than 2 years receive a further 1.0 point reduction. In the Italian Young Wheelchair Basketball Championship an additional class assigned 0.5 points, the Fascia Rossa (Red Belt) functional ability class, is also allowed to participate to improve inclusion. The Fascia Rossa players need to meet both the general IWBF eligibility criteria and bear a further permanent physical impairment resulting in a substantial loss of function in one or both upper extremities (e.g., tetraplegia). These players play with slightly modified on-court regulations (e.g. they are not required to bounce and they score one point if their shot hits the rim of the basket). At least one Fascia Rossa player per team must be on court during play.

In this study a national sample of younger Italian WB players competing in the Italian Young Wheelchair Basketball Championship was recruited with a fourfold aim: 1) To investigate the relationship between the players’ demographic as well as anthropometric and body composition variables, and performance using sport-specific field tests and game-related statistics; 2) To explore the relationship between performance in sport-specific field tests and game-related statistics; 3) To verify the relationship between the players’ characteristics and performance as well as functional ability classification; 4) To identify predictors of performance.

## Materials and Methods

### Participants

**Fig 1:**
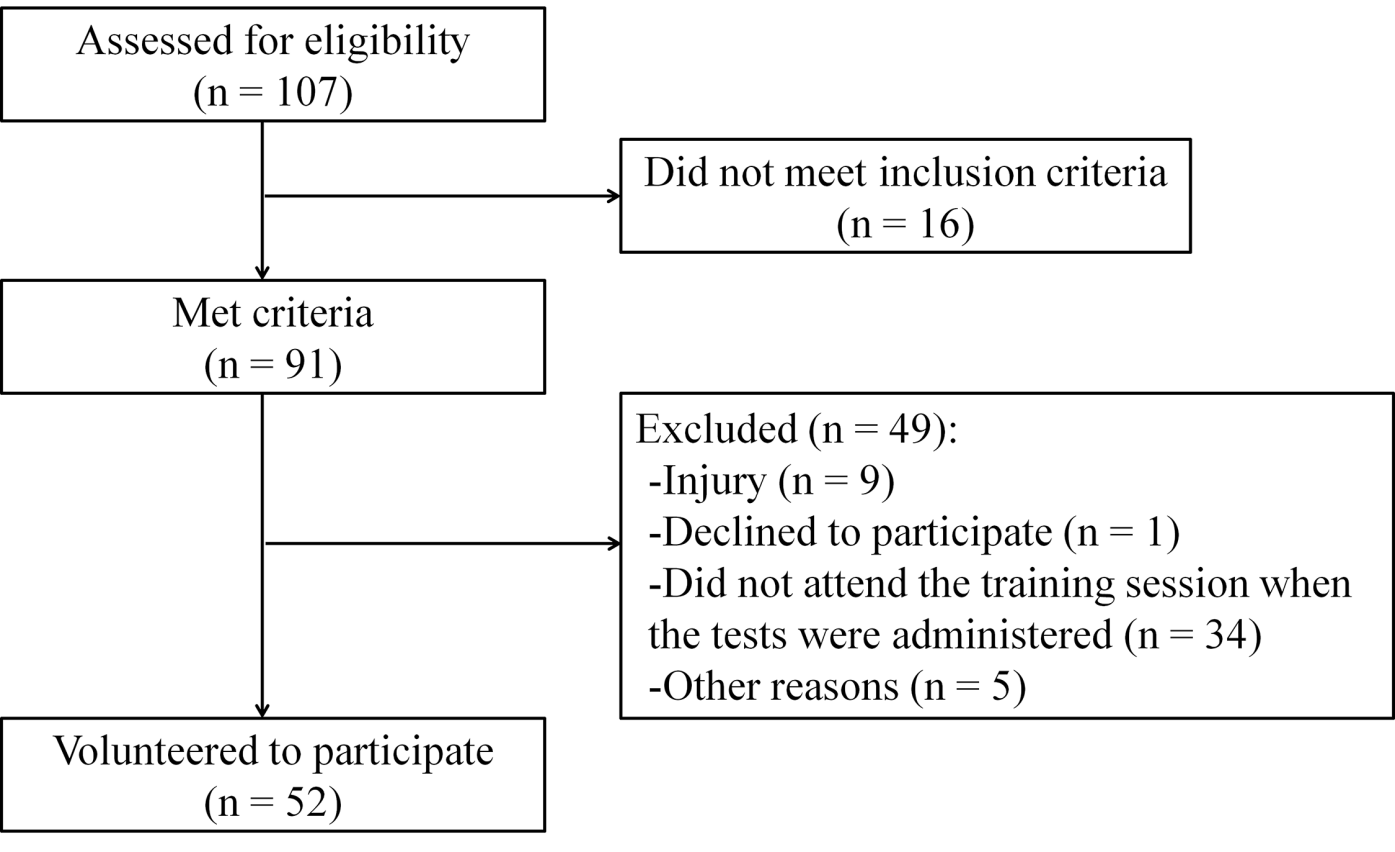
Recruitment flowchart.

In this cross-sectional study, the entirety of WB players participating in the 2013–2014 season of Italian Young Wheelchair Basketball Championship (n = 107) was assessed for eligibility (Fig 1). A total of 91 players fulfilled the inclusion criterion i.e., having played in more than two championship matches during the season; playing was considered being on court at least one time during a match. Age, sex and assigned functional points (0.5–4.5) were obtained for all participants from the database on the FIPIC website (www.federipic.it). Fifty-two players (57.1% of the eligible population) volunteered in this study; these played in eight out of nine Italian teams participating in the Italian Young Wheelchair Basketball Championship.

The young WB players participating in this study were male (n = 45) and female (n = 7) with mean age 18.1±4.6y and at least one complete season of WB experience (mean: 6.1±3.4y). All participants were actively training (estimated mean training time per week, 2.9±0.9h); they played 9.7±3.2 championship matches in the season. Disabilities comprised spinal cord injury (incomplete tetraplegia, n = 1; complete/incomplete paraplegia, injury level C6-T12, n = 6), other comparable neurologic disorders (n = 5), spina bifida (n = 17), cerebral palsy (n = 19), phocomelia (n = 2), lower extremity poliomyelitis (n = 2). All disabilities resulted in non-ability to play the running game, therefore matching the criterion set out by the IWBF (IWBF, 2011). The self-reported duration of injury was 13.1±9.4y. The distribution of participants across the assigned functional point range was as follows: Fascia Rossa (0.5 points), n = 19 (39% of total 0.5 points players in championship); 1.0 points, n = 8 (100% of total); 1.5 points, n = 8 (67% of total); 2.0 points, n = 3 (43% of total); 2.5 points, n = 5 (100% of total); 3.0 points, n = 5 (100% of total); 3.5 points, n = 2 (67% of total); 4.0 points, n = 2 (100% of total). The single 4.5 points player in the championship did not participate in this study. Within the scope of this study, players were grouped for comparative analysis into four functional ability Classes (A-D): Class A (n = 18) included 0.5 points Fascia Rossa players only; Class B was comprised of 1.0 and 1.5 points players (n = 16); Class C was comprised of 2.0 and 2.5 points players (n = 8); Class D was comprised of 3.0, 3.5 and 4.0 point players (n = 9). All participants signed informed consent form after they had been given full information about the purposes and the testing procedures of the study. Written informed consent was obtained from parent(s) in the case of underage subjects. The protocol conformed to the Declaration of Helsinki (revised in 2008). The Institutional Review Board at the University of Verona approved the study protocol. The study was conducted in the last forty days of the 2013/2014 competitive season (April-May).

### Assessment of performance

**Fig 2:**
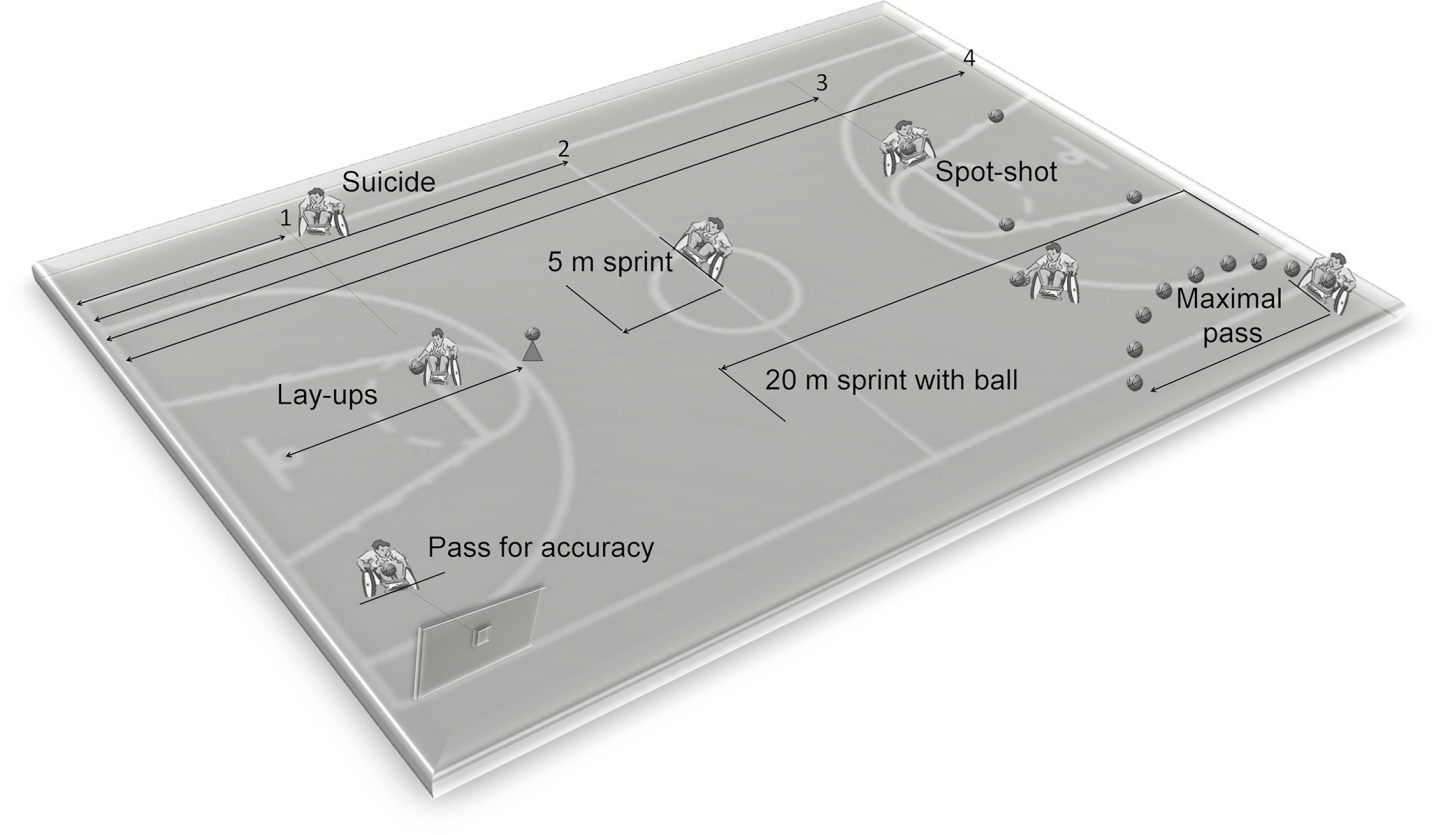
Layout of sport-specific field tests adopted in this study. See text for details.

The number of matches played by each player during the season was obtained from the score sheets (n = 53) of the whole championship (first and second championship phase, play off and final four). To respectively determine the quality of each player’s contribution to the match performance and the WB skills of the player, the game-related statistics obtained from the score sheets and the scores of sport-specific field tests were considered. The following game-related statistics were considered: 1) the number of free-throw points scored per match (FT), 2) the two- and three-point field-goals scored per match (FG), and 3) the total points scored per match (TP = FT+FG). The battery of sport-specific field test explored speed, ball handling, endurance, shooting, passing (accuracy and explosiveness) and included the following tests: 5m sprint, 20m sprint with ball, suicide, maximal pass, pass for accuracy, spot shot and lay-ups (Fig 2). All tests were performed according to de Groot et al. [7] and were modified to comply with the younger WB player’s capabilities as well as the relevant FIPIC rules. In particular, the height of the basket and the ball dimension were reduced as specified above, and the Fascia Rossa players were not required to bounce.

Field tests were performed subsequent to the team’s usual warm-up consisting of low to medium intensity wheelchair propulsion with and without the ball, acceleration and agility drills, shooting, passing, and stretching exercises. Field tests took place in the players’ play environment, on the basketball court in the gym of each team during one regular on-court training session. The sequence of tests was the same for all participants i.e., pass for accuracy, 5m sprint, 20m sprint with ball, maximal pass, lay-ups, spot shot and suicide; each participant was allowed a 2 min rest between the tests. During data collection, each participant used his own personal wheelchair and checked the tyre pressure in their chairs. For the 5m-sprint test, the player started from a stationary position, with the front wheels behind the start line and pushed for a distance of 5m as quickly as possible. The test was performed three times and the score was the average time of the three trials. For the 20m with ball test, the player started with a ball from a stationary position and pushed for a distance of 20m as fast as possible, adhering to the FIPIC rules for bouncing; the score was the time taken to complete the 20m. For the suicide test, the player positioned himself on the baseline, pushing first to the foul line (free-throw line) and back, then to the half line and back, then to the far foul line (free-throw line) and back, then to the far baseline and back. The total time to complete the test was the score. In the speed-related tests (5m sprint, 20m sprint and suicide), the player started sprinting on a starting sound. Time was manually recorded with a stopwatch starting when the front wheels crossed the start line and stopping when the front wheels crossed the finish line. For the maximal pass test, the player sat stationary with the front wheels behind the baseline, attempting to throw the basketball ball as far as possible. The distance between the baseline and where the ball first hits the floor was measured. For this test three trials were performed, and the score was the average. In the pass for accuracy test the player, from behind a 4m distance line, had to pass the basketball 10 times towards a 30cm square target (with a 2cm border) marked on the wall of the sports hall. The centre of the square was at 1.2m above the ground. Any form of pass was acceptable with the restriction that the ball may not bounce before hitting the target. Players scored 3, 1 or 0 points when they hit the target, the target border, or no target, respectively. The score was the sum of the points of the 10 passes (range: 0–30). For the lay-ups test, the players started with the basketball behind the 3-point line aiming to score as many lay-ups as possible within a minute. After each lay-up participants were asked to go back to the 3-point line and to pick up the ball from a cone. Depending on where the ball hits the scoring board, players scored 3 points (when the shot is a hit), 1 point (when the ball touches the ring but is not a hit) or 0 points (when the ball does not touch the ring at all). For the spot shot test, the player had to perform five shots from four positions around the lane (i.e. the area between the free-throw line and the base line), two at the top of the lane (left and right) and two at the base of the lane (left and right). Depending on where the ball hits the scoring board players scored 3 points (when the shot is a hit), 1 point (when the ball touches the ring but is not a hit) or 0 points (when the ball does not touch the ring at all). The score is the sum of the points of the 20 shots (range: 0–60).

### Anthropometry and body composition

**Fig 3:**
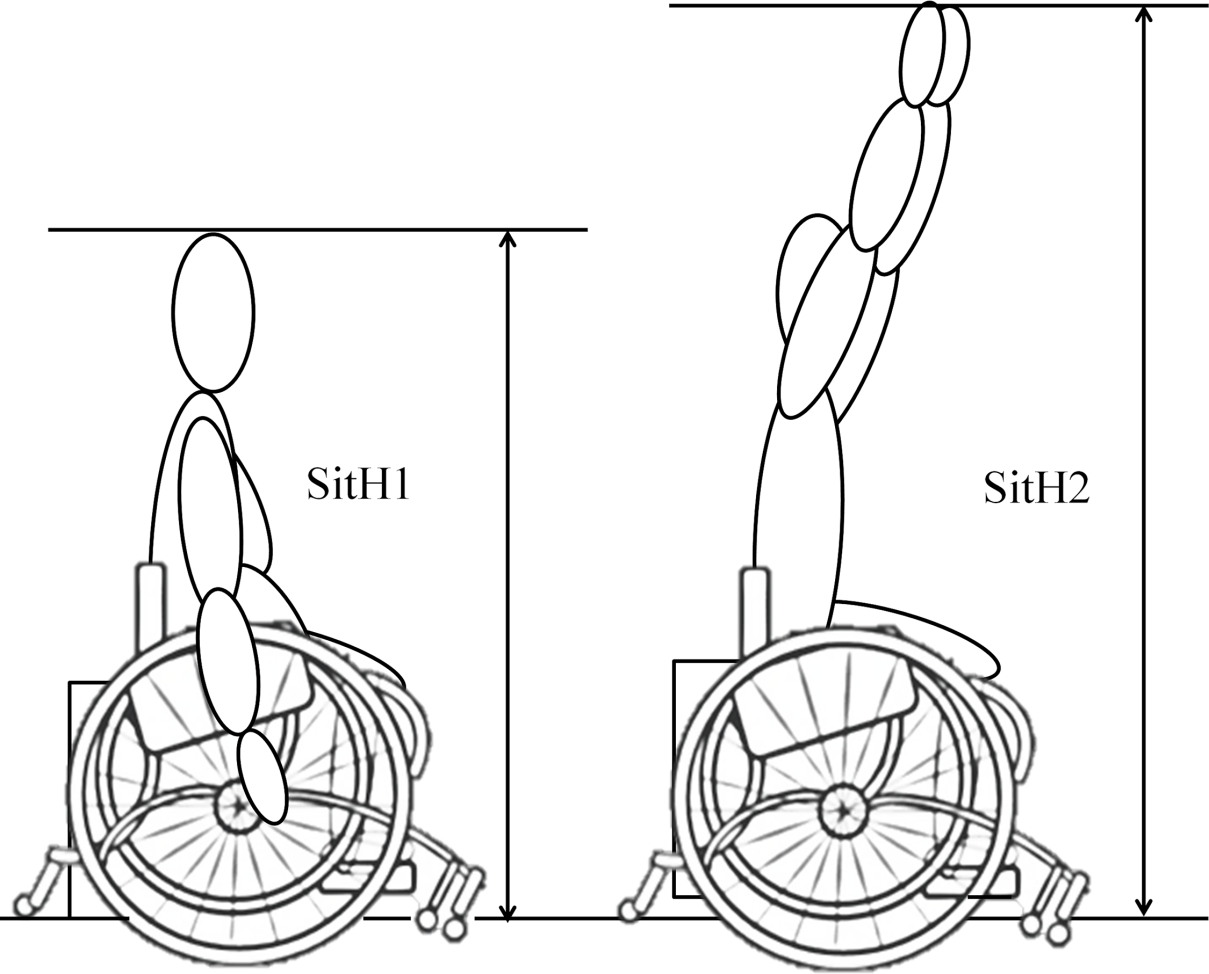
The two measurements of sitting height. SitH1: the vertical distance from the vertex of the head to floor; SitH2: the vertical grip reach from a seated position, was measured as the maximal distance from the tip of the dactylion III at the maximum to the floor, with the upper arms extended overhead as much as possible.

Body circumferences were measured with a fibreglass tape at the upper arm (relaxed), the forearm, the wrist and the waist. The following body dimensions were measured with a Harpenden anthropometer (Holtain Ltd., Crymych, Pembs. UK) according to conventional criteria and measuring procedures [15]: shoulder-elbow length, elbow-wrist length, thigh length, transverse chest width, anterior-posterior chest depth, elbow width and wrist width. Height is difficult to measure with accuracy in WB players because of the underlying pathology. In this study, the authors decided to adopt an ecological approach by measuring the height of the player on their own basketball wheelchair, assuming this is more representative of the real situation during play. Two measurements were taken: 1) the sitting height (SitH1), measured as the vertical distance from the vertex of the head to the floor; 2) the vertical grip reach from a seated position (SitH2), measured as the maximal distance from the tip of the dactylion III to the floor, with the upper arms extended overhead as much as possible (Fig 3). SitH1 and SitH2 were assessed in respect to the IWBF regulations for the ranges of wheelchair dimensions with maximum height set at 63 cm for players up to 3.0 points and 58 cm for 3.5–4.5 points players. Wheelchair height is calculated from the floor to the top of the cushion or the top of the seat platform where needed; measurements are taken with the front castor(s) in the forward driving position and free of the player. Skinfold thickness was measured to the nearest 0.1 mm with a Harpenden calliper (Gima, Milan, Italy) at the triceps, biceps, subscapular, and suprailiac sites according to standard procedures [15]. Duplicate readings were taken at each site, and the average of the two was the measure. If the two readings differed by more than 2 mm a third one was taken, and the closest two were averaged. The sum of the four skinfolds was used as an estimate of body density according to the general Durnin-Womersley equation [16] as previously reported in Paralympic sitting athletes [17]. Body density was then transformed into fat mass percentage (FM%) according to Siri [18]. All anthropometry and body composition measurements were taken on seated players by the same operator (VC).

### Statistical analysis

Data were assessed for normality with the Shapiro–Wilk test and log-transformed where necessary. Descriptive statistics (mean and standard deviation) was computed for all variables using standard procedures. Means difference was assessed with two-sample t-test (two-tailed). To assess the relationships between demographic (age, WB experience, and assigned functional points), anthropometric and body composition, and performance (game-related statistics and field tests) variables, the Pearson’s product-moment correlation coefficient (r) and the Spearman’s rho (ρ) were used for continuous and categorical variables, respectively. The strength of the correlation coefficient was considered small (0.00–0.30), moderate (0.31–0.49), large (0.50–0.69), very large (0.70–0.89), and almost perfect for assessing relationships (0.90–1.00) as per Hopkins [19]. In order to minimize Type I error associated with multiplicity of correlations in the same dataset, the Benjamini and Hochberg False Discovery Rate procedure was used to get corrected P-value (P_c_). For regression analysis, the participants were randomly assigned to two groups: a model development group (MD group, n = 35, 2/3 of sample) and a cross-validation group (CV group, n = 17, 1/3 of sample). The MD and CV groups were comprised of 13 and 6 Class A players, 10 and 6 Class B players, 6 and 2 Class C players, and 6 and 3 Class D players, respectively. In the MD group, separate stepwise multiple regression analyses (enter, F<0.05; remove, F>0.1) were run using demographic data, anthropometry and FM%, and field tests scores as independent variables to identify predictor(s) of individual of game-related statistics namely, TP, FT, and FG. Adjusted coefficients of determination (R^2^) and standard error of the estimate (SEE) were used to represent the goodness of the predictor model. Homoscedasticity of data was assessed by plotting the residuals of multiple regression analysis against the predicted values. The Durbin-Watson test, the variance inflation factor, the tolerance value, and the condition index were calculated to test collinearity. The developed regression models were then cross validated using the data from the CV group. Paired t-tests were performed to determine the difference between estimated game-related statistic and actual values. Reliability of data was assessed with the intraclass correlation coefficient (ICC; >0.75 = good reliability [20]), the Bland-Altman plot, and the standard error of measurement.

One-Way ANOVA was used to evaluate differences in the demographic, anthropometric, body composition, and performance variables (game-related statistics and field tests) of the four functional ability Classes; in case of significance, post-hoc comparisons were carried out with Bonferroni’s correction for multiple comparisons. The Levene’s test was performed to validate the application of ANOVA. Cohen’s f (f) and Cohen’s f^2^ (f^2^) were used to calculate the effect size in the ANOVA and the regression analysis, respectively. 95% confidence intervals (CI) were calculated for Cohen’s f and Cohen’s f^2^ statistics. According to Cohen’s guidelines [21], effect size values were interpreted as small (f = 0.1, f^2^ = 0.02), medium (f = 0.25, f^2^ = 0.15) and large (f = 0.4, f^2^ = 0.35) for ANOVA and for regression analysis effects, respectively. All analysis was performed with SPSS v. 16.0 (IBM Corp., Armonk, NewYork, USA). Post hoc statistical power of the sample was evaluated using G*Power Software 3.1 [22] on the basis of the observed effect sizes. The alpha value was set at 0.05.

## Results

### Participants

Age, games played in season, and game-related statistics (TP, FT, and FG) were similar in the sample of WB players recruited in this study (n = 52) and the eligible population (n = 91; P>0.05 for all, data not shown). Descriptive statistics for the four functional ability Classes (A-D) and the aggregate sample are summarized in Table 1.

**Table 1:** Biometric and performance variables in the four Classes (A-D) of younger WB players and the aggregate sample. Statistically significant P-values are in bold. Data are means±SD.

Variable	Aggregate sample	Functional ability class				ANOVA	
		A	B	C	D	F value	P value
	(n = 52)	(n = 19)	(n = 16)	(n = 8)	(n = 9)		
***Demographic***							
Age (y)	18.1±4.06	18.7±3.93	17.5±4.02	19.5±4.07	16.3±4.27	1.170	0.331
WB experience (y)	6.1±3.37	7.5±3.39	4.6±0.01	6.7±1.91	5.3±4.00	2.655	0.059
Games played in season (n)	9.6±3.16	9.0±3.32	8.8±3.67	12.1±1.13	10.3±1.87	2.651	0.059
***Circumferences***							
Upper arm (relaxed) (mm)	27.5±4.37	27.4±3.84	26.6±4.55	29.0±4.42	27.9±5.38	0.527	0.666
Forearm (mm)	25.0±3.13	24.4±2.65	24.4±2.95	26.6±3.24	25.8±4.03	1.383	0.259
Wrist (mm)	1.1±1.35	16.2±1.23	15.6±1.31	16.9±1.35	16.2±1.48	1.666	0.187
Waist (mm)	86.0±13.51	84.0±11.11	83.6±11.83	91.1±18.24	89.8±16.40	0.924	0.437
***Length/width/depth/breadth***							
Thigh length (cm)	36.0±5.11	35.7±5.81	35.2±4.55	36.9±2.77	37.5±6.36	0.461	0.711
Elbow breadth (cm)	6.3±0.55	6.3±0.65	6.2±0.51	6.6±0.45	6.2±0.42	0.970	0.415
Wrist breadth (cm)	5.0±0.45	5.1±0.52	4.9±0.44	5.0±0.34	5.2±0.32	1.255	0.300
Shoulder-elbow length (cm)	33.8±3.35	33.9±3.93	33.0±2.85	35.9±2.89	33.3±2.89	1.395	0.256
Elbow-wrist length (cm)	26.3±2.60	26.6±2.40	25.7±2.42	27.9±2.97	25.5±2.72	1.708	0.178
Transverse chest width(cm)	25.9±3.30	25.6±2.80	25.7±2.94	28.3±4.16	24.8±3.57	1.958	0.133
AP chest depth (cm)	18.9±2.79	18.8±1.85	18.1±3.13	19.9±3.47	19.5±3.19	0.968	0.416
SitH1 (cm)	127.0±10.76	125.0±10.35	124.2±10.97	129.6±4.90	134.1±12.73	2.114	0.104
SitH2 (cm)	164.6±14.30	157.9±12.81	162.8±10.86	171.7±9.25	175.4±18.58**	4.650	**0.006**
***Skinfolds and body composition***							
Subscapular (mm)	18.4±9.85	18.8±10.17	17.0±8.53	18.5±11.99	19.8±10.78	0.173	0.914
Triceps (mm)	17.3±7.97	16.6±7.85	17.1±7.80	16.9±7.90	19.6±9.51	0.291	0.831
Biceps (mm)	8.2±4.45	7.7±4.47	8.1±4.28	7.7±4.07	10.1±5.25	0.677	0.570
Suprailiac (mm)	21.0±9.28	19.6±8.15	19.7±9.47	21.6±9.46	25.7±10.98	1.009	0.397
Fat mass (%)	23.9±6.59	23.6±6.04	23.2±6.53	23.6±7.76	26.0±7.49	0.372	0.773
***Performance*: *game-related statistics***							
TP (n)	4.2±5.45	1.4±2.52	4.5±4.31	8.0±8.78*	7.8±6.14*	5.434	**0.003**
FT (n)	0.9±1.19	0.6±1.29	0.7±0.84	1.3±1.46	1.5±1.11	1.754	0.169
FG (n)	3.3±4.52	0.8±1.30	3.5±3.43	5.9±7.96**	5.6±5.16**	6.142	**0.001**
***Performance*: *sport-specific field test***							
5 m sprint (s)	2.7±0.66	3.2±0.65	2.4±0.44***	2.6±0.54*	2.2±0.19***	10.068	**<0.001**
20 m sprint with ball (s)	9.2±3.07	10.8±3.97	8.9±2.33	7.6±1.70	7.7±0.91	3.605	**0.020**
Suicide (s)	51.2±12.17	59.9±14.42	48.6±8.07*	45.1±6.59**	43.1±3.74***	7.529	**<0.001**
Pass for accuracy (n)	15.9±7.85	10.2±7.23	17.9±6.85**	17.5±4.57	23.1±4.73***	9.506	**<0.001**
Spot-shot (n)	19.4±11.83	12.3±8.94	20.7± 12.66	24.7±9.98*	27.3±10.32**	5.295	**0.003**
Lay-ups (n)	10.7±6.91	5.9±4.60	10.6±6.53	14.2±4.89**	17.9±5.64*** ^,^ ^	11.035	**<0.001**
Maximal pass (n)	7.8±3.40	5.8±2.23	7.4±2.46	9.8±3.56**	11.1±3.70*** ^,^ ^	8.860	**<0.001**

A, 0.5 functional points Fascia Rossa players; B, 1.0 and 1.5 functional points players; C, 2.0 and 2.5 functional points players; D, 3.0, 3.5 and 4.0 functional point players. TP, total points scored; FT, successful free-throws; FG, two- and three-point successful field-goals.

Post-hoc test (Bonferroni’s):

*, P<0.05 vs. A

**, P<0.01 vs. A

***, P<0.001 vs. A

^, P<0.05 vs. B

### Bivariate Correlation

The correlations between players’ assigned functional points as well as demographic, anthropometric, body composition variables, and performance (field tests and game-related statistics) are presented in Table 2. After Benjamini and Hochberg correction significant, moderate to large correlations were found between assigned functional ability class and all performance variables but FT. SitH2 showed significant, moderate to large correlation with all performance variables. Wrist width showed significant, moderate correlation with 20m sprint with ball and maximal pass. Forearm circumference significantly correlated with spot-shot, lay-ups, maximal pass, TP, FT and FG. Upper arm circumference, wrist circumference, shoulder-elbow length and SitH1 showed significant, moderate to large correlations with 5 out of 10 performance variables, while elbow width and elbow-wrist length significantly correlated with 3 out of 10 performance variables. Significant, moderate to large correlations were found between maximal pass and all anthropometric variables but the anterior-posterior chest depth. The strongest (r>0.50) correlations between individual anthropometric variables and performance were found between SitH2 and 5m sprint, 20m sprint with ball, spot shot, lay-ups, maximal pass, TP, and FG as well as forearm and wrist circumference, and maximal pass. No significant correlation was found between age, WB experience, skinfold thickness, FM%, and any performance variables.

**Table 2:** Bivariate correlation (Pearson’s two-tailed r for continuous variables; Spearman’s ρ for categorical variables) between age, WB experience, functional points class, anthropometric and body composition variables, and performance variables (field tests [T1-T7] and game-related statistics) in younger WB players (n = 52). Significant correlations are in bold.

		T_1_	T_2_	T_3_	T_4_	T_5_	T_6_	T_7_	TP	FT	FG
Age	r	0.08	0.09	0.15	0.08	0.15	0.02	0.10	-0.06	-0.15	-0.08
	P_c_	*0*.*749*	*0*.*724*	*0*.*491*	*0*.*724*	*0*.*491*	*0*.*907*	*0*.*660*	*0*.*820*	*0*.*497*	*0*.*896*
WB experience	r	0.13	0.07	0.20	-0.12	0.11	-0.08	0.14	0.01	0.00	-0.04
	P_c_	*0*.*548*	*0*.*779*	*0*.*196*	*0*.*627*	*0*.*631*	*0*.*724*	*0*.*536*	*0*.*940*	*0*.*978*	*0*.*935*
Functional point class	r	**-0.53**	**-0.48**	**-0.54**	**0.59**	**0.49**	**0.62**	**0.57**	**0.48**	0.33	**0.51**
	P_c_	***<0*.*001***	***0*.*004***	***<0*.*001***	***<0*.*001***	***<0*.*001***	***<0*.*001***	***<0*.*001***	***0*.*003***	*0*.*063*	***<0*.*001***
Upper arm circumference	r	-0.14	-0.14	-0.19	0.10	**0.40**	0.25	**0.47**	**0.38**	**0.41**	**0.35**
	P_c_	*0*.*536*	*0*.*524*	*0*.*366*	*0*.*660*	***0*.*019***	*0*.*182*	***0*.*008***	***0*.*024***	***0*.*019***	***0*.*041***
Forearm circumference	r	-0.25	-0.24	-0.19	0.20	**0.47**	**0.38**	**0.61**	**0.46**	**0.41**	**0.44**
	P_c_	*0*.*179*	*0*.*196*	*0*.*345*	*0*.*307*	***<0*.*001***	***0*.*024***	***<0*.*001***	***0*.*008***	***0*.*015***	***0*.*008***
Wrist circumference	r	-0.16	-0.27	-0.09	0.16	**0.38**	0.31	**0.53**	**0.39**	**0.39**	**0.37**
	P_c_	*0*.*458*	*0*.*159*	*0*.*722*	*0*.*457*	***0*.*024***	*0*.*086*	***<0*.*001***	***0*.*023***	***0*.*024***	***0*.*031***
Waist circumference	r	0.03	-0.05	-0.06	-0.02	0.26	0.11	**0.36**	0.25	0.18	0.25
	P_c_	*0*.*904*	*0*.*852*	*0*.*820*	*0*.*921*	*0*.*173*	*0*.*660*	***0*.*041***	*0*.*195*	*0*.*401*	*0*.*185*
Thigh circumference	r	-0.27	-0.14	-0.19	0.17	0.28	0.33	**0.34**	0.21	0.13	0.22
	P_c_	*0*.*146*	*0*.*537*	*0*.*363*	*0*.*438*	*0*.*146*	*0*.*057*	***0*.*049***	*0*.*284*	*0*.*538*	*0*.*263*
Elbow width	r	-0.23	-0.22	-0.10	0.17	**0.37**	**0.35**	**0.48**	0.33	0.31	0.31
	P_c_	*0*.*212*	*0*.*265*	*0*.*668*	*0*.*429*	***0*.*031***	***0*.*044***	***<0*.*001***	*0*.*064*	*0*.*084*	*0*.*082*
Wrist width	r	-0.34	**-0.45**	-0.27	0.29	0.33	0.26	**0.40**	0.27	0.26	0.26
	P_c_	*0*.*051*	***0*.*008***	*0*.*146*	*0*.*107*	*0*.*064*	*0*.*164*	***0*.*019***	*0*.*146*	*0*.*163*	*0*.*167*
Shoulder-elbow length	r	-0.19	**-0.34**	-0.17	0.22	**0.34**	0.26	**0.44**	**0.38**	0.23	**0.40**
	P_c_	*0*.*366*	***0*.*049***	*0*.*429*	*0*.*268*	***0*.*049***	*0*.*163*	***0*.*008***	***0*.*028***	*0*.*232*	***0*.*023***
Elbow-wrist length	r	-0.17	-0.15	-0.14	0.03	0.27	0.16	**0.45**	**0.39**	0.24	**0.41**
	P_c_	*0*.*407*	*0*.*492*	*0*.*536*	*0*.*904*	*0*.*155*	*0*.*441*	***0*.*008***	***0*.*023***	*0*.*196*	***0*.*019***
Transverse chest width	r	-0.11	-0.18	-0.21	0.09	0.28	0.19	**0.38**	0.24	0.16	0.24
	P_c_	*0*.*650*	*0*.*401*	*0*.*300*	*0*.*722*	*0*.*127*	*0*.*757*	***0*.*028***	*0*.*210*	*0*.*457*	*0*.*196*
AP chest depth	r	0.00	-0.05	-0.05	-0.03	0.20	0.05	0.31	0.20	0.12	0.22
	P_c_	*0*.*978*	*0*.*852*	*0*.*852*	*0*.*903*	*0*.*310*	*0*.*864*	*0*.*077*	*0*.*307*	*0*.*628*	*0*.*270*
SitH1	r	-0.26	**-0.37**	-0.25	0.24	**0.37**	**0.38**	**0.49**	0.34	0.10	**0.38**
	P_c_	*0*.*173*	***0*.*034***	*0*.*182*	*0*.*196*	***0*.*031***	***0*.*024***	***<0*.*001***	*0*.*051*	*0*.*668*	***0*.*024***
SitH2	r	**-0.44**	**-0.51**	**-0.41**	**0.41**	**0.56**	**0.53**	**0.69**	**0.60**	**0.34**	**0.63**
	P_c_	***<0*.*001***	***<0*.*001***	***0*.*019***	***0*.*019***	***<0*.*001***	***<0*.*001***	***<0*.*001***	***<0*.*001***	***0*.*049***	***<0*.*001***
Subscapular skinfold	r	0.10	-0.09	-0.06	-0.09	0.08	-0.04	-0.01	0.07	0.15	0.05
	P_c_	*0*.*683*	*0*.*722*	*0*.*824*	*0*.*704*	*0*.*759*	*0*.*879*	*0*.*969*	*0*.*776*	*0*.*492*	*0*.*864*
Triceps skinfold	r	0.04	-0.06	-0.11	-0.10	0.08	0.04	-0.06	0.02	0.18	-0.03
	P_c_	*0*.*896*	*0*.*820*	*0*.*636*	*0*.*670*	*0*.*749*	*0*.*882*	*0*.*824*	*0*.*931*	*0*.*401*	*0*.*907*
Biceps skinfold	r	0.02	-0.08	-0.17	-0.04	0.11	0.07	-0.05	0.03	0.19	-0.02
	P_c_	*0*.*913*	*0*.*749*	*0*.*413*	*0*.*896*	*0*.*631*	*0*.*762*	*0*.*864*	*0*.*907*	*0*.*357*	*0*.*926*
Suprailiac skinfold	r	-0.03	-0.25	-0.15	0.09	0.12	0.04	0.13	0.03	-0.02	0.04
	P_c_	*0*.*907*	*0*.*195*	*0*.*491*	*0*.*724*	*0*.*596*	*0*.*896*	*0*.*567*	*0*.*907*	*0*.*907*	*0*.*896*
% Fat mass	r	0.03	-0.15	-0.11	-0.05	0.11	0.03	0.03	0.05	0.09	0.03
	P_c_	*0*.*903*	*0*.*497*	*0*.*631*	*0*.*864*	*0*.*631*	*0*.*907*	*0*.*904*	*0*.*864*	*0*.*724*	*0*.*903*

T_1,_ 5 m sprint; T_2,_ 20 m sprint with ball; T_3,_ Suicide; T_4,_ Pass for accuracy; T_5,_ Spot-shot; T_6,_ Lay-ups; T_7,_ Maximal pass; TP, total points scored; FT, successful free-throws; FG, two- and three-point successful field-goals. P_c,_ Benjamini and Hochberg corrected P-value.

The correlations between sport-specific field tests scores and game-related statistics (TP, FT, and FG) are reported in Table 3. After Benjamini and Hochberg correction, all field tests showed moderate to very large correlation (r = 0.36–0.78; P_c_, 0.008-<0.001) with TP, FT and FG. The 5 m sprint, 20m sprint with ball, suicide, and pass for accuracy tests showed lower correlations (r = 0.36–0.54) with game-related statistics in comparison with the spot-shot, lay-ups, and maximal pass tests (r = 0.57–0.78). Variables showing significant correlation with individual game-related statistics (TP, FT, FG) were used in regression analysis.

**Table 3:** Pearson’s two-tailed r between game-related statistics and field tests in younger WB players (n = 52). All correlations are statistically significant.

		T_1_	T_2_	T_3_	T_4_	T_5_	T_6_	T_7_
**TP**	r	-0.52	-0.45	-0.54	0.51	0.69	0.68	0.77
	P_c_	<0.001	0.001	<0.001	<0.001	<0.001	<0.001	<0.001
**FT**	r	-0.42	-0.36	-0.46	0.41	0.63	0.63	0.57
	P_c_	0.002	0.008	0.001	0.003	<0.001	<0.001	<0.001
**FG**	r	-0.51	-0.45	-0.53	0.51	0.67	0.65	0.78
	P_c_	<0.001	0.001	<0.001	<0.001	<0.001	<0.001	<0.001

TP, total points scored; FT, successful free-throws; FG, two- and three-point successful field-goals.

T_1,_ 5 m sprint; T_2,_ 20 m sprint with ball; T_3,_ Suicide; T_4,_ Pass for accuracy; T_5,_ Spot-shot; T_6,_ Lay-ups; T_7,_ Maximal pass. P_c,_ Benjamini and Hochberg corrected P-value.

### Multiple regression analysis

The MD and CV group were similar for all measured variables by two-sample t test but for the upper arm, the forearm and the waist circumference (P = 0.025, 0.021 and 0.043, respectively). In the MD group, entering anthropometric, body composition, and sport-specific field test scores in stepwise multiple regression analysis yielded statistically significant models for any game-related statistics (TP, FT, FG; P<0.001 for all): The three models were:

1. TP = -5.589 + 0.885 (maximal pass) + 0.301 (lay-ups); adjusted R^2^ = 0.589, SEE = 3.84
2. FT = -2.722 + 0.103 (lay-ups) + 0.093 (upper arm circumference); adjusted R^2^ = 0.416, SEE = 0.99
3. FG = -5.232 + 1.093 (maximal pass); adjusted R^2^ = 0.577, SEE = 3.28

**Fig 4:**
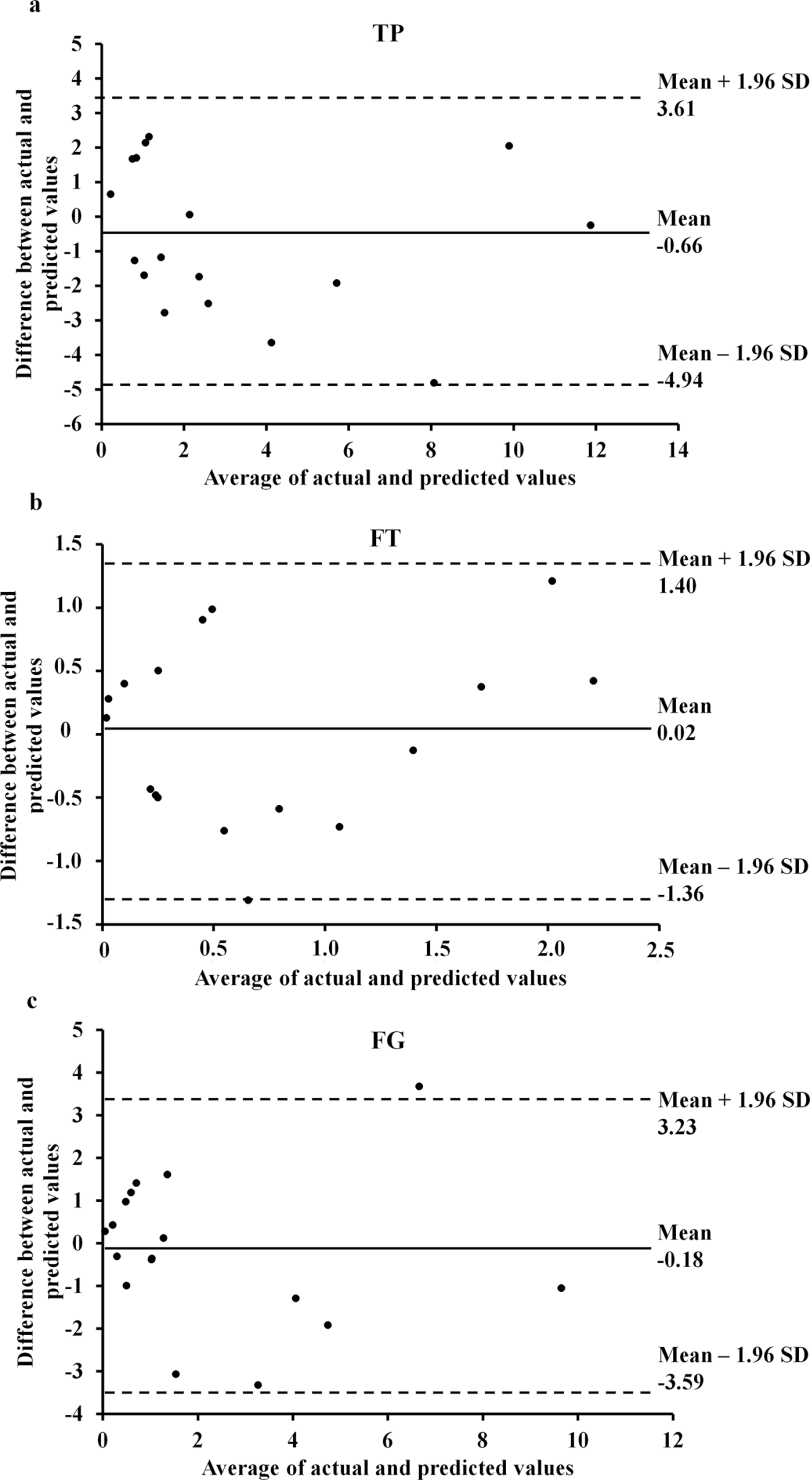
Bland-Altman plots of the differences between actual game-related statistic and predicted values. (a): mean points scored per match (TP); (b): mean free-throw points scored per match (FT); (c): mean two- and three-point field-goals scored per match (FG).

For all models, the Durbin-Watson test, the variance inflation factor, the condition index, and tolerance value was <2.3, <1.8, <15.8 and >0.5, respectively, showing that they were robust to collinearity. The f^2^ was 1.58 [95% CI, 0.75–3.90], 0.82 [95% CI, 0.29–2.06], and 1.36 [95% CI, 0.60–3.48] for model 1, 2, and 3, respectively. Post hoc power analyses revealed that the statistical power for regression analyses in this study exceeded 0.99, suggesting good model sensitivity to Type II error as well as that the models were adequately powered to detect the true effect of the predictor variables. In the CV group, the estimated game-related statistics were not significantly different from actual values (TP, t = 0.227; FG, t = 0.675; FT, t = 0.926; P = N.S. for all, two-tailed t-test). The correlation between estimated and actual values was significant (TP, r = 0.87, P<0.001; FT, r = 0.70, P = 0.02; FG = 0.83, P<0.001); the ICC and the standard error of measurement were 0.923, 0.824 and 0.907, and 0.60, 0.68, and 1.70 for TP, FT and FG, respectively. The Bland-Altman plot with 95% confidence limits (Fig 4) showed good agreement between estimated and actual values with a very limited number of outliers. Upper and lower limits of agreement were 1.46 and 5.09, 0.37 and 1.09, 0.83 and 3.58 for TP, FT, and FG, respectively.

### Functional ability Class

Results of ANOVA in the four functional ability classes (A-D) are reported in Table 1. No significant difference was found for age, WB experience, games played in season, FT as well as FM% and all anthropometric variables with the exception of SitH2 (f, 0.54 [95% CI, 0.40–0.68]). Significant differences were found for all sport-specific field tests (f, 0.47 [95% CI, 0.34–0.60]-0.83 [95% CI, 0.64–1.02]) as well as TP and FG (f, 0.58 [95% CI, 0.43–0.73] and f, 0.61 [95% CI, 0.46–0.76], respectively). For all significant differences, the effect size (f) was >0.47, indicating large effect. Post-hoc tests showed that that most performance outcomes were significantly worse in Class A vs. Class D and, to a more limited extent, Class C and Class B. However, post hoc power analyses revealed that there was an 82 to 99% chance of detecting a very large effect size (f >0.5) significant at the 0.05 level (two tailed). Power analysis with power (1 – β) set at the recommended 0.80 level (9) and α = 0.05 (two-tailed) indicated the sample size would have to increase up to approximately 1096, 180 and 76 subjects to detect a small, medium and large effect size, respectively.

## Discussion

### Main findings

In this work, a majority (57.1%) of younger Italian WB players was recruited to investigate the relationships between the players’ characteristics, performance, and functional ability classification. The number of matches played in the season as well as the game-related statistics were similar in the sample (n = 52) and the eligible population (n = 91), showing that the former is representative of the latter in terms of sport exposure and performance. Results demonstrate the following points: 1) Several players’ anthropometric variables, and Class significantly correlate with performance outcomes, but WB experience, skinfolds and FM% do not; 2) Within performance outcomes, game-related statistics and sport-specific field tests all show significant correlation with each other; 3) Selected anthropometric variables and/or sport-specific field tests are able to significantly predict game-related statistics; 4) The current functional-point based classification system reflects to a limited extent the actual differences in performance of younger WB players.

Being based on a large, representative sample of players and a battery of sport-specific field tests, this work offer normative data for anthropometry and performance of younger WB players which may be of use to coaches and trainers to develop training programs and identify training priorities.

### Players’ characteristics and performance

In our sample, no correlation was found between FM%, skinfold thickness, and performance variables (game-related statistics and sport-specific field tests). This may come as some surprise, because a low FM% is considered a key factor of performance in running basketball and, in general, able-bodied sports [23]. In fact, fat mass acts as a dead weight to be moved, and also affects energy expenditure. However, due to the fact that WB players carry their body weight in a wheelchair during play and do not have to jump, they may not be hindered by extra body weight as in running basketball. They may even gain some advantage from a more stable posture due to extra body weight when throwing the basketball. These aspects may reduce the direct impact of body fat on performance. Nevertheless, in this work we used an anthropometric equation developed in able-bodied subjects to calculate FM%; given the changes in body composition and fat distribution associated with spinal cord injury or other comparable neurologic disorders after spinal cord injury [24,25], specific anthropometric equations derived from wheelchair athletes should be developed to provide more accurate assessment of FM% and its relationships with performance in WB players. Preliminary results from our laboratory showed that FM% is underestimated in spinal cord-injured athletes by about 3% using the Durnin and Womersley equation [16] when dual-energy X-ray absorptiometry is the reference.

Results of the present study show that assigned functional points and sitting height are the main younger WB players’ characteristics associated with performance in terms of both sport-specific field tests score and game-related statistics (Table 2). This suggests that severity of impairment and height from floor are strongly involved in WB performance. Notably, nor age nor WB experience significantly associated with performance in younger WB players, indicating that physical maturation and specific sport practice have limited relevance in WB proficiency. The relationship between functional ability class and sitting height is complex and deserves discussion. Height is difficult to measure in individuals with physical impairments such as paralysis, spasticity, bone deformities etc. These impairments may make standing upright or maintaining an erect sitting or lying flat problematic or impossible [26,27]. In several published studies researchers have relied on height that was ascertained from self-reports [28,29] or height measured in the supine position [30,31]. An alternative to measuring height is the use of proxy measures of body segment lengths. However this method may be questioned due to the impossibility to obtain standard criteria for measurements in this population. Moreover, all of these methods aim to calculate heights or measurements that are not related to the actual height of the player during play. In this work, the authors were interested in finding a height measurement which is suitable for all players while giving a proxy of their actual height during play; accordingly, we measured SitH1 and SitH2 (see Methods) as more representative of the real situation during play. Results showed that SitH2 is especially relevant to performance, because it correlated more strongly and with a larger number of sport-specific field test scores and game-related statistics than SitH1; therefore, SitH2 will be given special attention in the following discussion. In our sample, SitH1 and SitH2 significantly correlated with height-related variables namely, shoulder-elbow (r = 0.54, and r = 0.56, respectively), elbow-wrist (r = 0.41 and r = 0.48, respectively), and thigh length (r = 0.62 and r = 0.52, respectively) (P<0.001 for all), indicating that young WB players with greater SitH1 or SitH2 are also taller. Actually, in able-bodied subjects, standing height is proportional to sitting height [32]. In running basketball, even if a player’s height is fixed and can be partially balanced with jumping abilities, the relationship between height of both adult and young players and their success in running basketball has been accepted for a long time [33,34]. The results of the present study suggest that the player's height is important in WB as well. This is supported by previous findings showing that the height of a discus or shot at release and the standing height of the athlete are associated with throwing performance in seated athletes with disabilities [35,36]. However, SitH2 is reached when the body is extended with shoulder, elbow, and wrist angles approximating 180° (Fig 3), such a position depending on the extent of motion of the segments. Some spastic disorders are often associated with reduced range of movement in one or more joints; moreover, any damage to the musculature of the trunk due to spinal lesions, would reduce the subject’s ability to elevate their arm. Further, sitting height is affected by the player’s sitting position. Players with most activity limitation (e.g., paraplegics) gain stability and balance with a deeper-seated position on the basketball wheelchair. This deeper position is formed with the inclination of the seat producing an angle between the trunk and the thighs of less than 90° and may vary with the degree of disability, thereby affecting sitting height. Accordingly, Class A players (including players with more severe impairment) had lower mean values for SitH2 in comparison to all other Classes, and functional point class positively correlated with SitH2. In summary, the subject’s height and degree of impairment interplay in determining SitH1 and SitH2.

The association of increasing sitting height with better game-related statistics in WB has several explanations. First, it is reasonable to assume that SitH2 influences the height of ball release during a shot. Actually, increasing the ball release height augments the chance for a shot to be successful [37], and the shot in is considered the most important ability in WB performance [11,38]. Second, greater SitH2 would be relevant in recovering the ball by steals, blocking shots or opponent’s turnovers as well as receiving a pass or ball recovery after a bounce off the backboard. For example, Wang and colleagues [4] showed in a large sample of male and female WB players participating in 2000 Sydney Paralympic Games that the sitting height, measured as the distance between the greater trochanter to the acromion while the participant is sitting on their own wheelchair, significantly contributes to average rebounds per game. Further, recovering the ball by steals, blocked shots or opponent’s turnovers is the most important variable in both genders’ unbalanced games in WB [9], similar to running basketball [12]. However, it should be kept in mind that successful shots (and, in turn, TP), rebounds, assists, steals, blocks and so on are influenced by several technical and tactical abilities of players that have not been considered in the present study.

Intriguingly, SitH2 significantly correlated with the players' score in all sport-specific field tests, also (Table 2), indicating that SitH2 is associated with sport-specific motor abilities in WB. It has been shown that in handrim-propelled wheelchairs a positive relationship exists between shoulder-to-seat height (i.e. the vertical distance between the acromion and the seat) and the mechanical efficiency [39]. Accordingly, further investigation is required to explore the relationship between SitH2 and different temporal-spatial, kinematic, muscle and/or kinetic parameters involved in wheelchair propulsion in order to evaluate its role in terms of mechanical efficiency.

Several upper body anthropometric variables were positively and significantly correlated with one or more performance outcomes in our sample. In particular, larger wrist circumference was associated with better performance in some sport-specific field tests and all game-related statistics, followed by upper arm lengths/circumferences. In WB, almost no force from the lower extremities can be used when shooting, passing, laying up the basketball, and propelling the wheelchair. Accordingly, the force generated from the trunk and the upper arm must be transferred to the forearm through elbow extension [40] to get the ball to the basket or to a teammate or to propel the wheelchair, ultimately relying on the joint action of wrist flexion/extension in terms of range of motion and muscle strength. Moreover, longer upper arm lengths would facilitate passing and shooting by giving the players, whose body is constrained in the wheelchair, more room to move the ball while receiving or making a pass in WB games. Our findings are supported by data from Wang et al. [4], showing that the wrist joint is important to WB performance and that wrist muscle strength may be more important than that in other joints for wheelchair manoeuvring and ball control.

### Field tests and game-related statistics

In our sample, all field tests showed statistically significant, moderate to very large correlation with game-related statistics. This suggests that increasing sport-specific abilities associate with better on court performance. In particular, the maximal pass test showed the largest positive correlations with TP and FG, followed by spot shot and lay-up. It is conceivable that the ability to shoot at the basket from a wider range of distances and with greater accuracy positively impacts on game scoring. Speed- (5m sprint, 20m sprint with ball) and endurance- (suicide) related tests showed weaker, albeit significant, association with game-related statistics, indicating that the player’s ability to move and sustainably propel the wheelchair is also involved in game scoring.

### Predictors of performance

The maximal pass and lay-ups tests predicted about 59% of the total variance for TP, the lay-ups test and upper arm circumference predicted about 42% of the total variance for FT, and the maximal pass predicted about 58% of total variance for FG. It should be taken into account that there is an inherent heterogeneity in the reference population i.e., wheelchair athletes, making the development of very accurate predictive model cumbersome. However, the ability of the maximal pass and lay-ups tests in predicting TP outperformed that of three range of motion factors (59 vs. 41% of explained variance) as shown by Wang et al. [4] in adult WB players. Reliability of the current models is also supported by cross validation analysis showing that predicted values in the CV group are similar to the actual values by t test; moreover, correlation (Pearson r and ICC) and Bland-Altman analyses showed good agreement between predicted and actual values; the mean difference was negligible for FT; for FG and TP the mean difference was negative, suggesting a slight underestimation of performance (Fig 4). Upper and lower limits of agreement were moderately wide, indicating that differences between measures for some individuals were large; however, the confidence interval for the mean difference includes zero and, accordingly, the mean game-related statistics values (predicted and actual) are similar. Overall, the current models should be able to reasonably predict game-related statistics in other WB populations. Interestingly enough, younger WB players in our sample showed game-related statistics and sport-specific test scores comparable with that of adult WB players [4,7], suggesting that the current models could be of general use in WB players. However, caution should be taken when applying these models to an individual player’s performance because results in all field performance tests but the 5 m sprint and the suicide tests were obtained using the special FIPIC regulations (e.g. the height of the basket, the ball size and the Fascia Rossa functional ability class; see Introduction for details), and all the three models show relatively large SEEs and do not predict a substantial proportion of variance in game-related statistics.

### Functional ability class and performance

When performance (sport-specific field tests and game-related statistics) was compared in the four functional ability Classes (A-D), results suggest that assigned functional points affect WB proficiency. This is consistent with the positive, significant correlation between functional ability class and any performance variables (Table 2). However, post hoc analysis only showed limited class-class difference, which was more evident for Class A and D i.e., those classes including players with the lower and higher functional ability, respectively. The paucity of significant differences in performance for players belonging to intermediate functional ability Classes (B, C) and Class D may indicate that the current classification system does not entirely match the actual functional potential of the members of some functional ability classes. The notable heterogeneity of athletes within each Paralympic sport and the complexity of the Paralympic classification process have led to a number of researchers questioning the classification systems and their validity for athletes with disabilities among the Paralympic sports [3,41,42]. Accordingly, the International Paralympic Committee has mandated the development of ‘evidence-based classification systems through research’ [43]. In countries where initiatives specific for young wheelchair basketball players are already present, the main aim is that of recreation and socialisation but this does not detract from the fact that competition is available and should be regulated to create a fair and unbiased structure for all those who participate. Participation in wheelchair basketball by young male and female players should be encouraged and facilitated promoting an appropriate evidence-based classification of athletes on the basis of their functional and performance abilities. The current results represent a first step toward such an aim in Italy.

The present study has several limitations that should be acknowledged. Considering functional ability classification, it should be noted that the half point functional classes were not considered and that 3.0, 3.5 and 4.0 points players merged in one Class (D) due to the low number of participants. This may have obscured some differences between functional ability classes. Accordingly, more extensive studies recruiting a larger number of younger WB players are required to confirm conclusions presented here. Second, the sample size was only adequate to detect a large effect size level with an acceptable power, although we were able to test 57.1% of the eligible population. However, it should be considered that a highly restricted and extremely heterogeneous sample is an inherent limitation when studying disabled athletes. Third, the overall in-season playing time was not available for most players, thereby preventing the relationship between game-related statistics and the actual on-court time to be explored. Finally, the absolute number of female players was low in this study, despite we recruited 7 out of 13 females playing in the Italian Young Wheelchair Basketball Championship. In this championship males and females play together, raising the question as to whether any differences exist in terms of skill proficiency between male and female players in the same IWBF functional point class. Due to the small number of young female players participating in the Italian wheelchair basketball championship and the heterogeneity of their activity-limiting impairments this matter could not be explored in the present study.

## Conclusion

This study investigated the physical characteristics of a nation-wide sample of younger wheelchair basketball players in relation with performance in sport-specific tests and game-related statistics. The results of the present study support the following conclusions: 1) Age, WB experience and FM% do not influence WB performance; 2) Sitting height positively contributes to WB performance; 3) Maximal pass, lay-ups and upper arm circumference, alone or in combination with each other, significantly predict game point scoring and should be carefully considered in young WB physical and technical training plans; 4) Large overlapping in both sport-specific field tests performance and game-related statistics is present in younger WB players belonging to functional ability Class B, C, and D.

Taken together, our findings suggest that the classification system of younger WB players would benefit from closer scrutiny of objectively measured functional ability. Future research in a larger number of younger WB players is needed to explore gender differences, position specificity, body composition and other physiological and biomechanical factors which have the greatest effect on young WB performance in terms of training strategies and classification systems.
